# Identification and expression analysis of putative chemoreception genes from *Cyrtorhinus lividipennis* (Hemiptera: Miridae) antennal transcriptome

**DOI:** 10.1038/s41598-018-31294-9

**Published:** 2018-08-28

**Authors:** Gui-Yao Wang, Jing-Lei Zhu, Wen-Wu Zhou, Su Liu, Quais Md Khairul, Naved Ahmad Ansari, Zeng-Rong Zhu

**Affiliations:** State Key Laboratory of Rice Biology; Key Laboratory of Molecular Biology of Crop Pathogens and Insects, Ministry of Agriculture; Institute of Insect Sciences, Zhejiang University, Hangzhou, Zhejiang 310058 China

## Abstract

*Cyrtorhinus lividipennis* Reuter (Hemiptera: Miridae) is an important egg predator of planthoppers which are destructive rice pests. The chemosensory genes in the mirid antennae play important roles in mating and prey-seeking behaviors. To gain a better understanding of the olfaction of *C. lividipennis*, we sequenced the antennal transcriptomes of the predator to identify the key olfaction genes. We identified 18 odorant binding proteins (OBPs), 12 chemosensory proteins (CSPs), 1 Niemann-Pick C2 protein (NPC2), 15 odorant receptors (ORs), 6 ionotropic receptors (IRs), 3 gustatory receptors (GRs) and 3 sensory neuron membrane proteins (SNMPs). Quantitative real-time PCR results showed that the relative transcript levels of three ClivORs (*ClivOR6*, *7* and *14*) in the female antennae were 3 to 6 folds higher than that in the male antennae, indicating these genes were more related to oviposition site selection. The relative transcript levels of *ClivCSP8* and *ClivOR11* were 2.6 and 2.7 times higher in the male antennae than that of the female, respectively, indicating that these genes might be involved in mate searching. Moreover, the responses of ds*orco* treated predators to volatiles emitted from infested rice were significantly reduced, indicating these volatiles might serve as crucial cues in the host searching of *C. lividipennis*.

## Introduction

Natural enemies of herbivorous insects often depend on volatile chemical cues to locate their concealed prey in the complex environment^1^. For example, some parasitoid species are attracted by herbivore-induced plant volatiles during the foraging process^2^. *Cyrtorhinus lividipennis* Reuter (Hemiptera: Miridae) is an important egg predator of planthoppers and leafhoppers which are destructive rice pests in Asia^3–5^. Some studies have reported the role of rice volatiles in regulating the behavior of natural enemies^6^. *C. lividipennis* were found to be attracted by volatiles emitted from herbivore-infested plants, suggesting that olfaction played an essential role in their prey search^6,7^. The antenna, covered with different types of chemosensory sensilla, is the specialized organ for olfaction in insects^8^. Olfactory perceptions of Hemipteran species, such as *Tropidothorax elegans*^9^ and *Apolygus lucorum*^8,10^, rely largely on chemosensory genes. Identification of chemosensory genes in *C. lividipennis* can provide better understanding of how the predator utilizes chemical cues in their search behavior in agricultural systems^11^.

Chemical cues are transformed into electrical signals by olfactory receptor neurons (ORNs) housed within the sensilla and then these signals are transmitted to the brain to finally elicit distinct behaviors^8,12–14^. The key olfactory proteins involved in the perception of odorants in insects are odorant-binding proteins (OBPs), chemosensory proteins (CSPs), Niemann-Pick C2 protein (NPC2), odorant /ionotropic receptors (ORs and IRs), gustatory receptors (GRs) and sensory neuron membrane proteins (SNMPs)^5,15–19^.

OBPs and CSPs are small soluble proteins that are highly abundant in the chemosensilla lymph of insects^20^. The two soluble proteins can transport hydrophobic odorants through the sensillar lymph to activate membrane-bound ORs^15,21^. A typical OBP (generally 135–220 amino acids) contains six conserved cysteine residues paired into three disulfide bridges. The OBPs undergo ligand-induced conformational shifts that trigger the firing of ORNs^22,23^. Some studies have showed that OBPs play different roles by binding with various odorants^24^. For instance, CquiOBP1 was reported to bind with a oviposition pheromone in *Culex pipiens quinquefasciatus*^25^. CsupOBP8 was found to be associated with the recognition of plant volatiles in *Chilo suppressalis*^26^ and OBP99a in *Bactrocera dorsalis* was found to be involved in the selection of ovipostion hosts^24^. The CSP (normally 100–120 residues) has four conserved cysteines that form two disulfide bridges and bear no sequence similarity to OBPs^20,27,28^. The CSPs have been reported to perform different functions, such as leg regeneration and development^5,29–31^. Liu *et al*. also found that CSP4 acted as surfactant in the proboscis of two *Helicoverpa* species^32^. CSPs were also reported to act as carriers for visual pigments in insects^33^.

NPC2 are highly divergent between species in arthropods^19,28^. These proteins share some structrual and functional characteristics with OBPs and CSPs^19^. Some studies have shown that NPC2 proteins act as carriers for semiochemicals and other hydrophobic compounds^19,34^.

Insect ORs belong to the seven-transmembrane domain (TMD) protein family with a reversed topology of having intracellular N-terminus^35,36^. The conventional insect ORs show great diversity in the DNA sequence levels, which reflect their rapid evolution^37^. The odorant receptor coreceptor (Orco), highly conserved among insect species, forms ligand-gated ion channels with other ORs to enhance odorant responsiveness^15,38–40^. In fact, Orco can form heterodimeric complexes with conventional ORs that are responsible for binding to diverse odorants^41^. Disruption of the Orco function can dramatically impair olfactory behavior responses in various insect species, such as *A. lucorum*, *Harpegnathos saltator* and *Locust amigratoria*^10,42,43^. IRs are relatives of ionotropic glutamate receptors (iGluR) which represent elements for sensing both external and internal chemical cues^44,45^. IRs are supposed to form two or three subunits coexpressed in the same neuron^35,45^. They are divided into two major groups, the conserved “antennal IRs” and “divergent IRs”^22,46,47^. Some GRs are coexpressed in chemosensory neurons which are involved in carbon dioxide detection^48^. However, GRs are mainly expressed in gustatory receptor neurons in taste organs, which can detect bitter compounds, sugars and contact pheromones^22^.

Finally, the SNMPs are proteins of the CD36 family that are crucial for pheromone perception^18^. Insects generally have two SNMP subfamilies (SNMP1 and SNMP2). The SNMP1 subfamily was found to be associated with pheromone detection in *Drosophila melanogaster* and several lepidopteran species^18,49^. However, the function of SNMP2 remains poorly understood^21^.

In this study, we performed Illumina sequencing to identify putative chemosensory genes in the adult *C. lividipennis* antennae. We identified 18 OBPs, 12 CSPs, 1 NPC2, 15 ORs, 6 IRs, 3 GRs and 3 SNMPs in the transcriptome dataset. The expression patterns of these genes in different tissues were examined by quantitative real-time PCR (qRT-PCR). We further explored the foraging behavior of the predator *C. lividipennis* by silencing *orco* in a laboratory experiment.

## Results

### Illumina sequencing and sequence assembly

A total of 60,658,602 and 53,853,286 clean reads were obtained from the *C. lividipennis* male and female antennal transcriptome, respectively. The clean reads are available in the NCBI Sequence Read Archive (SRA accession: SRP128761). The combined assembly of all clean reads generated 62,637 unigenes with a mean length of 1,401 bp, and N50 of 2,338 bp and N90 of 588 bp (Supplementary Table S1).

### Functional annotation

**Figure 1:**
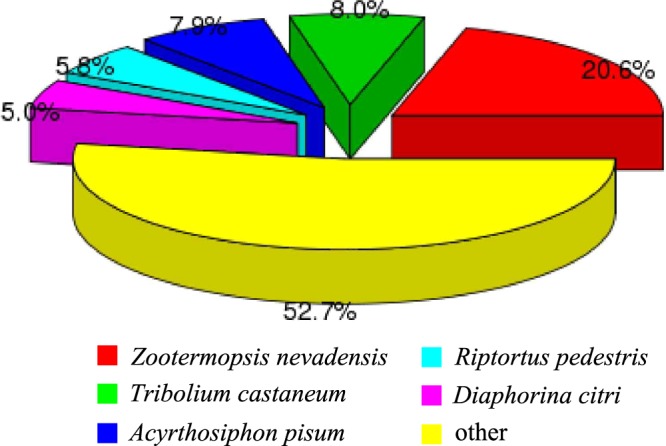
Species distribution of the *C. lividipennis* antennal transcriptome unigenes based on the results of BLASTX search. Different colors represent different species.

**Figure 2:**
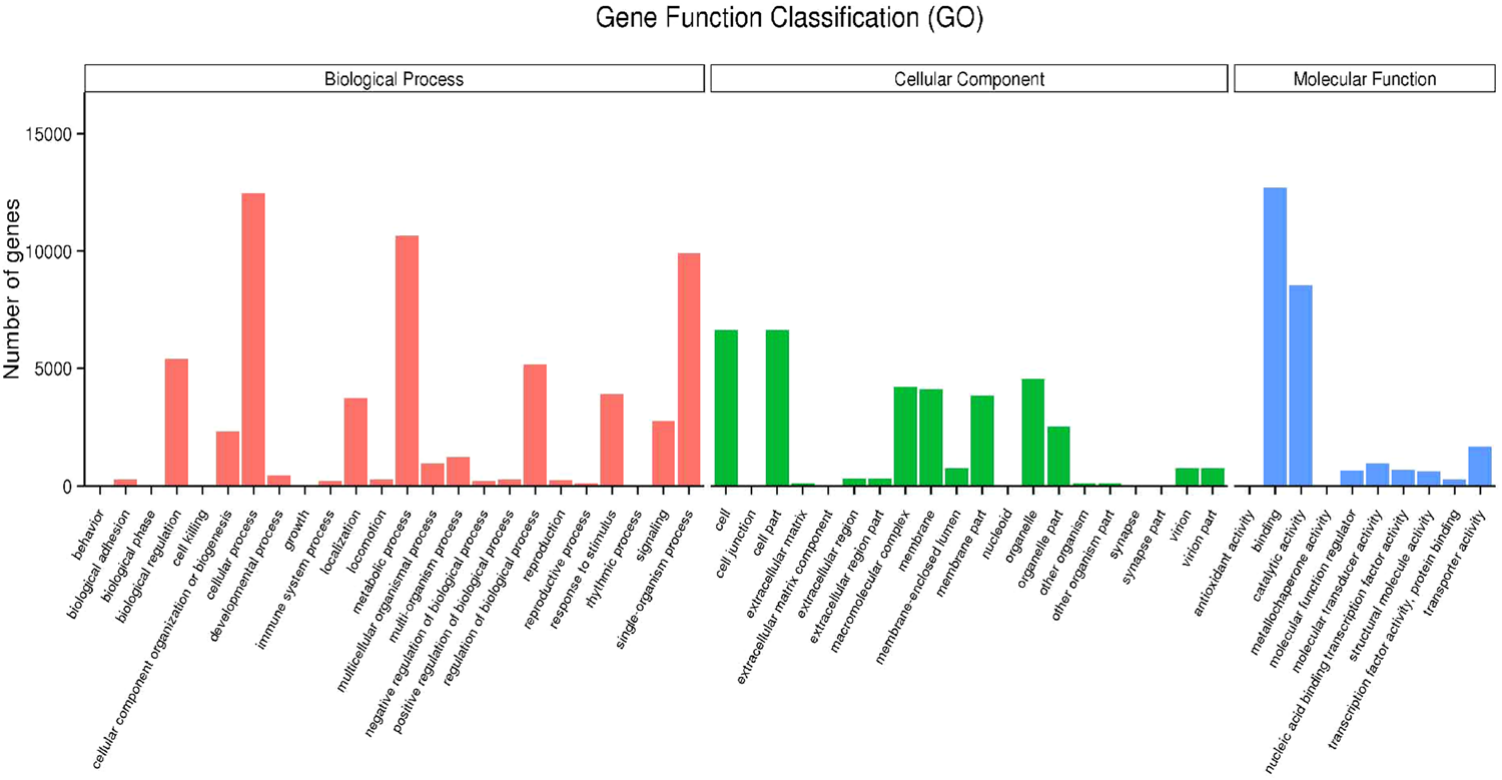
Gene ontology classifications of *C. lividipennis* antennal transcriptome unigenes. The left y-axis denote the number of genes in the category.

28,147 (44.93%) unigenes were annotated in at least one of the databases. The numbers of unigenes annotated to different databases are shown in Table 1. The largest numbers of unigene annotations are deposited in NR database (23,113, 36.89%). BLASTX homology searched against the NCBI-Nr database showed that the *C. lividipennis* antennal unigenes were best matched to sequences from *Zootermopsis nevadensis* (20.6%), followed by *Tribolium castaneum* (8.0%), and *Acyrthosiphon pisum* (7.9%) (Fig. 1). Gene Ontology (GO) assignments were used to classify the *C. lividipennis* antennal transcriptome unigenes into three main functional groups: biological processes, cellular components and molecular functions (Fig. 2). Among the 62,637 unigenes, approximately 34.66% (21,712) of the unigenes could be assigned to GO terms (Table 1). Cellular process (12,485, 20.49%) was the most prevalent terms in the category of biological processes. The cellular components were equally dominated by cell part (6,625, 18.53%) and cell (6,625, 18.53%). Binding (12,700, 48.55%) represented the most abundant GO terms in the molecular function category.

**Table 1:** Summary of unigenes annotations.

Annotation databases	Number of unigenes	Percentage (%)
NR Annotation	23,113	36.89
NT Annotation	4,663	7.44
Swissprot Annotation	18,370	29.32
Pfam Annotation	21,615	34.5
GO Annotation	21,712	34.66
KOG Annotation	11,630	18.56
Annotated in all databases	2,259	3.66
Annotated in at least one database	28,147	44.93

NR: non-redundant protein; NT: nucleotide sequences; Pfam: Protein family; GO: Gene Ontology; KOG: euKaryotic Ortholog Groups.

### Identification of candidate OBPs

In total, we identified 18 putative OBPs from the *C. lividipennis* antennal transcriptome. Of these genes, 10 were previously reported in the whole body transcriptome of adult *C. lividipennis* (Genbank No. KY462016-KY462025). All of the other 8 newly identified OBP sequences (named *ClivOBP11-18*) had full-length ORFs with predicted signal peptide. The results of BLASTX are shown in Supplementary Table S2. All the ClivOBPs were best matched to known Miridae OBPs. The identities of three pairs of OBPs were higher than 70%: *ClivOBP15* and *LlinOBP3* (98%), *ClivOBP17* and *LlinOBP11* (75%), *ClivOBP18* and *LlinOBP18* (72%). The remaining pairs showed identities ranging from 44 to 67%. Multiple sequence alignments of the newly identified *C. lividipennis* OBPs indicated that the eight ClivOBPs belongs to classic OBPs (carried six conserved cysteine residues) (Supplementary Fig. S1A)^20,50^.

The phylogenetic tree was constructed to reveal the relationships of ClivOBPs to those of other hemipteran species, including three Miridae (*A. lucorum*, *Lygus lineolaris* and *Adelphocoris lineolatus*) and three Delphacidae species (*Nilaparvata lugens*, *Sogatella furcifera* and *Laodelphax striatella*). The tree revealed that ClivOBPs spread across several branches. Several ClivOBPs (*ClivOBP12, 13, 14, 16* and *18*) were clustered with AlucOBPs in one subbranch (Supplementary Fig. S2).

### Identification of candidate CSPs and NPC2

Twelve putative CSPs and one NCP2 were identified in the *C. lividipennis* antennal transcriptome. Among them, five CSP sequences were also reported in the whole body transcriptome of adult *C. lividipennis* (Genbank No. KY462026-KY462030). The remaining seven ClivCSPs were named from *ClivCSP6* to *ClivCSP12*. The results of BLASTX are shown in Supplementary Table S2. All the deduced ClivCSPs sequences had full-length ORFs with the conserved four cystine residues (Supplementary Fig. S1B). *ClivCSP7* and *ClivCSP12* showed the highest identities to *AsutCSP4* (81%) and *AlinCSP12* (80%) respectively, while *ClivCSP9* and *ClivCSP10* showed identities <57% to other known CSPs.

A phylogenetic analysis was performed to show the relationships among ClivCSPs and CSPs from other hemipteran species, including two Miridae (*A. lucorum* and *A. lineolatus*) and two Delphacidae species (*N. lugens* and *L. striatella*). The ClivCSPs phylogenetic tree showed that ClivCSPs spread across several branches. Some ClivCSPs (ClivCSP9, ClivCSP12) were closely related to NlugCSPs (Supplementary Fig. S3).

### Identification of chemoreceptor genes

**Figure 3:**
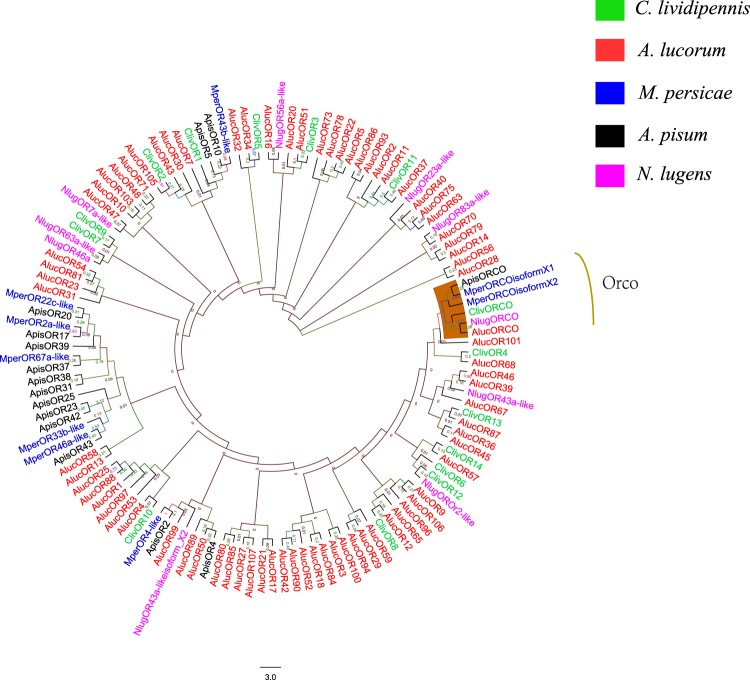
Phylogenetic analysis of ORs from five hemipteran insects. Cliv, *Cyrtorhinus lividipennis*; Aluc, *Apolygus lucorum*; Mper, *Myzus persicae*; Apis, *Acyrthosiphon pisum*; Nlug, *Nilaparvata lugens*.

A total of 15 candidate ORs, 6 candidate IRs and 3 putative GRs were identified in the *C. lividipennis* antennal transcriptome. The results of BLASTX are shown in Supplementary Table S3. All of the ORs contained full-length ORFs ranged from 314 to 490 amino acid residues with 4–8 transmembrane domains. Seventeen ClivORs shared 28–77% sequence identities with the ORs in *A. lucorum*. The *C. lividipennis* Orco sequence showed the highest identity (89%) to the *A. lineolatus* Orco. Of the 6 candidate IRs, four unigenes were predicted to have full-length ORFs with a least one TMD. The ClivIRs shared 22–57% sequence identities with other insect IRs (Supplementary Table S3). We performed the phylogenetic tree to better understand the relationships of the ClivOR proteins with ORs in other hemipteran species, including one Miridae (*A. lucorum*), two Aphididae (*Myzus persicae* and *A. pisum*) and one Delphacidae species (*N. lugens*).The phylogenetic tree revealed that the *C. lividipennis* Orco was clustered with Orcos in other insect species (Fig. 3).

### Identification of candidate SNMPs

We found two subfamilies of SNMPs in *C. lividipennis* (1 ClivSNMP1 and 2 ClivSNMP2, Supplementary Table S3). The ClivSNMPs showed 35–51% identities with other insect species. In addition, the three ClivSNMPs had two transmembran domains.

### Sex-specific expression of *C. lividipennis* chemoreception genes

**Figure 4:**
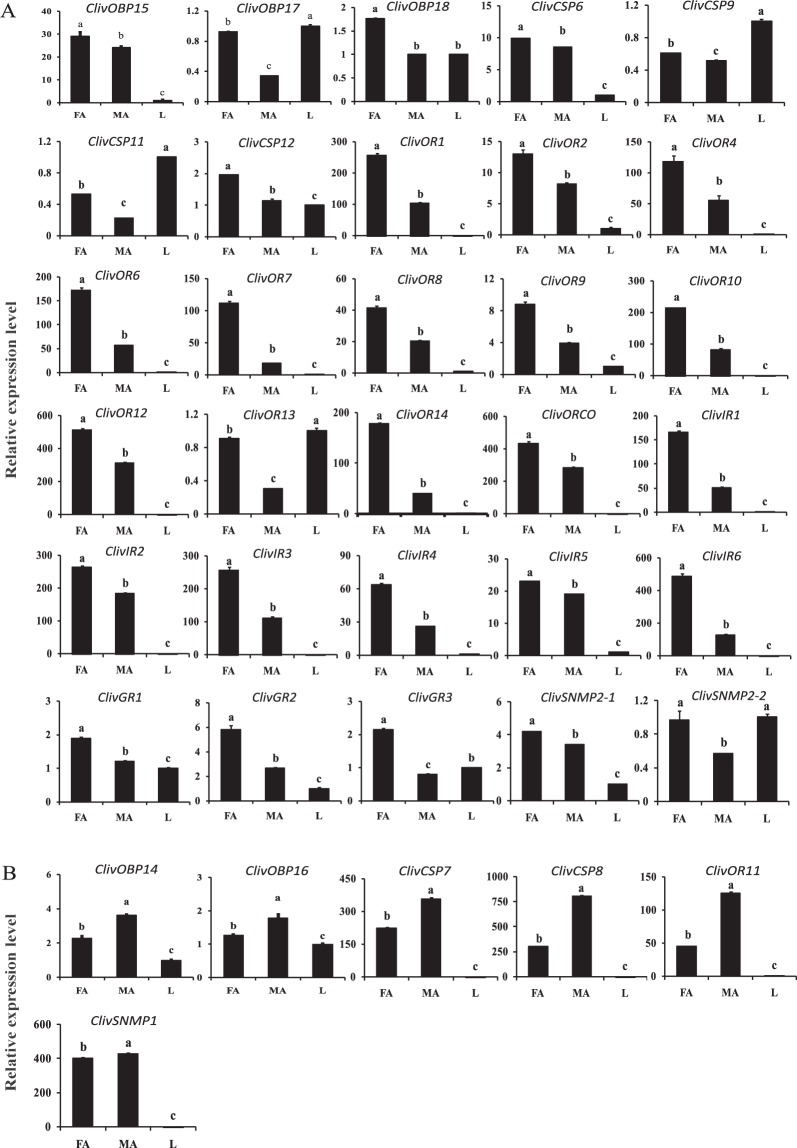
Sex-specific expression of *C. lividipennis* chemoreception genes. (**A**) The female-dominantly expressed olfactory genes. (**B**) The male-dominantly expressed olfactory genes. Gene expression patterns in antennae were normalized relative to legs (male and female mixture). Data were presented as the mean of three replicates (n = 3) ± SE. Different lower cases indicate significant differences (p < 0.05). FA: female antennae, MA: male antennae, L: legs.

Results of the qRT-PCR assays indicated that three ClivOBPs (*15*, *17*, *18*), four ClivCSPs (*6*, *9*, *11*, *12*), twelve ClivORs (*1*, *2*, *4*, *6–10*, *12*, *13*, *14* and *orco*), ClivIRs (*1–6*), three ClivGRs (*1–3*) and two ClivSNMPs (*ClivSNMP2-1* and *2-2*) were more highly expressed in the female antennae than in the male. In particular, the relative transcript levels of three ClivORs (*6*, *7* and *14*) in the female antennae were 3 to 6 folds higher than in the male (Fig. 4A). Besides, two ClivOBPs (*14*, *16*), two ClivCSPs (*7*, *8*), *ClivOR11* and *ClivSNMP1* were highly expressed in the male antennae. Among these genes, the relative expression levels of *ClivCSP8* and *ClivOR11* were 2.6 and 2.7 times higher in the male antennae than that of the female, respectively (Fig. 4B). In addition, three ClivOBPs (*11*, *12*, *13*), *ClivCSP10*, *ClivNPC2* and two ClivORs (*3*, *5*) were expressed in both the male and female antennae with similar transcript accumulations (Supplementary Fig. S4).

### Responses of *C. lividipennis* to different odors after silencing *orco* gene

**Figure 5:**
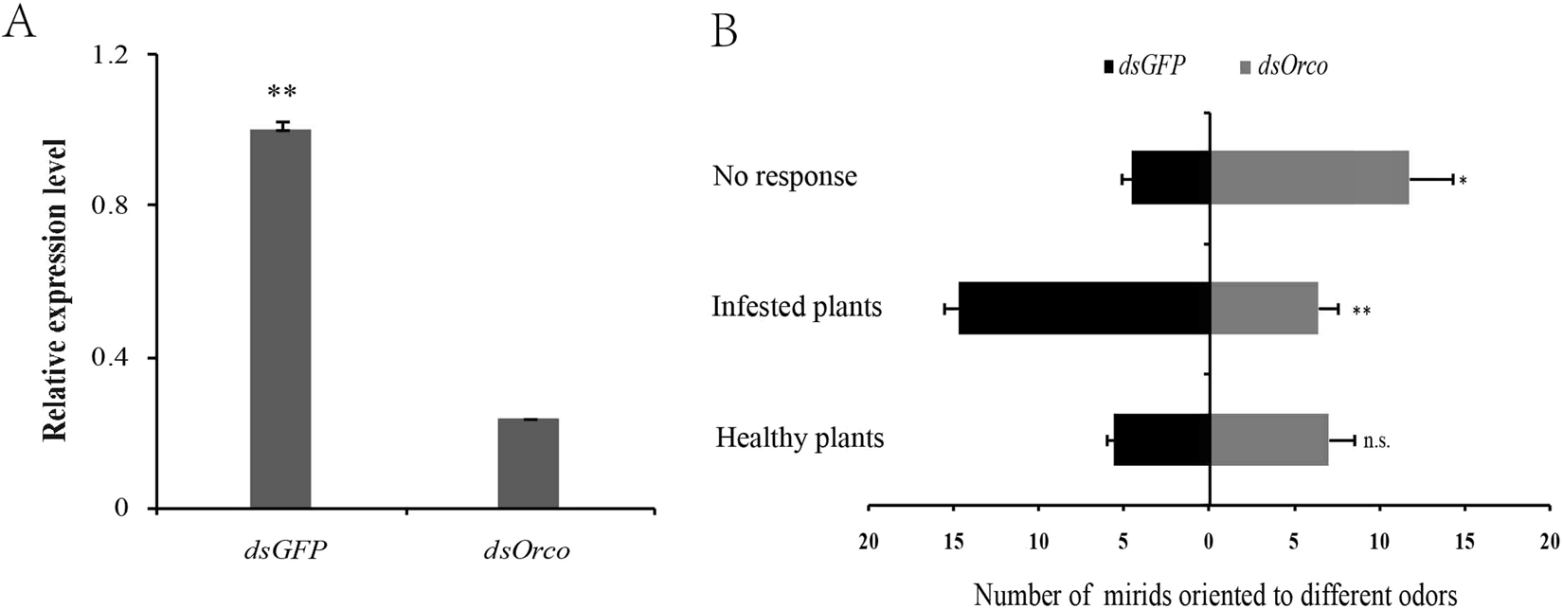
Responses to different odor sources by *C. lividipennis* after dsRNA silencing treatment. (**A**) Relative transcript accumulation of *orco* after RNAi were quantified by qRT-PCR. (**B**) Responses of *C. lividipennis* to different odor sources after ds*GFP* and ds*orco* treatment. Infested plants, healthy plants denote volatiles emitted by gravid female-damaged rice seedlings and healthy rice seedlings respectively. *, **, and n.s. refereed to the difference between two treatments (ds*GFP* and ds*orco*) is significant (*p* < 0.05), highly significant (*p* < 0.01), and not significant (*p* > 0.05) (*t*-test), respectively.

We used RNAi to investigate the functions of *orco* in the foraging behavior of *C. lividipennis*. ds*orco* treated predators showed an approximately 80% decrease in the transcripts of *orco* compared to ds*GFP* treatments (Fig. 5A). These ds*orco* and ds*GFP* treated mirids were further used for olfactory response study.There were significant differences in the foraging behavior between the ds*orco* and the ds*GFP* treated insects. The responses of the ds*orco* treated predators to volatiles emitted by infested plants were significantly lower than those of the ds*GFP* treated individuals (t = 5.9285, df = 7.189, p < 0.01). The number of mirids showing no response was higher under the ds*orco* treatment than the ds*GFP* treatment (t = 2.6848, df = 4.451, p < 0.05). No obvious differences of ds*orco* and ds*GFP* treated predators in response to volatiles released by healthy plants were observed, indicating that some other genes might be involved in olfactory responses (t = 0.8801, df = 4.587, p = 0.4225) (Fig. 5B).

## Discussion

*C. lividipennis* is one of the most important natural enemies of planthoppers in Asian rice fields^3^. However, studies on the insect predator’s olfactory system are scarce^36^. It has been shown that *C. lividipennis* rely on herbivore-induced rice volatiles to identify eggs of *N. lugens*^6^ and the antennae are the main olfactory organs for this insect. The *C. lividipennis* antennal transcriptome dataset would be able to provide a better molecular understanding of its olfactory systems that can improve the effectiveness of the predator in biological control.

In this study, we identified 58 putative olfactory genes (18 OBPs, 12 CSPs, 1 NPC2, 15 ORs, 6 IRs, 3 GRs and 3 SNMPs) based on the transcriptome analysis of male and female antennae of *C. lividipennis*. The number of OBPs identified in *C. lividipennis* was more than those in *Sitobion avenae* (13 OBPs), *N. lugens* (11 OBPs) and *A. pisum* (15 OBPs)^50–52^, but less than those in *Tessaratoma papillosa* (33 OBPs) and *A. lucorum* (38 OBPs)^8,46^. The number of the CSPs in *C. lividipennis* was close to the previous findings in other hemipteran insects such as *A. pisum* (13CSPs), *S. furcifera* (9 CSPs) and *N. lugens* (17CSPs)^50,52,53^. In addition, we identified 24 chemosensory receptors (15 ORs, 6 IRs and 3 GRs) in *C. lividipennis*, which was fewer than that in other insect species such as *Anoplophora chinensis* (44 ORs, 23 IRs and 19 GRs)^13^ and *Cylas formicarius* (54 ORs, 15 IRs and 11 GRs)^54^. The number of identified olfactory genes varied in different species, which might be the limitation of the Illumina sequencing methods and depth^55^. The transcriptome data may only represent part of expressed chemosensory genes in the cell but not those genes with low transcript abundance or no expression^56,57^.

The chemosensory genes of *C. lividipennis* showed various similarities with the genes from other hemipteran species, which might be caused by their different host preferences^53^. In the phylogenetic trees, the OBPs and CSPs in *C. lividipennis* clustered with olfactory genes in other species (like *A. lucorum*, *L. lineolaris*), which suggested that these genes might have similar functions in general odorant perception^21^. In addition, most of the ClivORs were clustered with ORs in *A. lucorum*, which might be involved in the detection of host plant volatiles^8^.

Several chemosensory genes were reported to have sex biased transcript accumulation in other insects, such as in *T. papillosa* and *A. lucorum*^8,46^. Both mating and feeding behavior strongly rely on chemical cues. Some studies showed that the chemosensory genes were associated with the perception of plant volatiles and sex pheromones^26,58^. Our study showed that the expression patterns of several chemosensory genes in *C. lividipennis* had sexual differences. The genes more highly expressed in the female antennae (*ClivOR6, 7* and *14*) might encode proteins involved in oviposition site selection^6,46^. Some genes (such as *ClivOBP14* and *ClivOR11*) were male-dominantly expressed, indicating the preferential functions in the detection and discrimination in mate searching^21,59^. There was no significant difference in the expression of the other genes (such as *ClivOBP11* and *ClivOR3*), which might have more basic functions in binding general volatiles^21,51^.

Orco is the highly conserved olfactory co-receptor that plays important roles in OR-mediated chemosensation^43^. Orco has been identified in most insect species, including *D. melanogaster*, *A. pisum* and *B. dorsalis*^41,60–62^. In the study, the ClivOrco grouped with other Orcos, indicating that Orco was highly conserved within these hemipteran species. The *orco* gene does not function directly in odor recognition but rather encodes the obligate co-receptor of all ORs, which significantly impacts olfaction^10^. Some studies showed that the disruption of *orco* resulted in reductions in olfactory sensitivity in *Drosophila* and other insects^42,43,63^. It was also reported that *orco* mutations impaired social behavior plasticity, reproduction and development of ORNs in ants^38,43^. In this study, the transcripts of *Clivorco* were much more abundant compared to conventional ORs, which was consistent to the findings from *Chrysoperla sinica*^36^. After silencing of *orco* gene, around half of the treated predators showed no response to the volatiles emitted by healthy plants or infested plants. The formation of the OR-Orco dimers could be disrupted in the ds*orco* treated mirids, which could lead to the reduction of OR-mediated chemosensation^10^. Thus, many ds*orco* treated predators could not respond to volatiles emitted from the healthy or infested plants. In addition, ds*orco* treated *C. lividipennis* showed lower responses to the volatiles emitted by BPH-infested rice plants compared with the ds*GFP* treated insects, indicating the *orco* gene may play crucial roles in the host searching of *C. lividipennis*. Our study provides a foundation for further investigations into the functions of the specific chemosensory genes associated with different olfactory cues. EAG (or single sensillum recording) test of ds*orco*-treated and ds*GFP*-treated predators might provide solid conclusions about behavioral responses of the predator to plant volatiles^64^.

## Materials and Methods

### Insects rearing

The *C. lividipennis* individuals used in this study were originally collected from rice fields in Zi Jin Gang campus of Zhejiang University in Hangzhou, China. The laboratory colony was reared in a climate room at 26 ± 1 °C and 70% relative humidity under a photoperiod of 16:8 h light: dark for several generations. The fifth instar nymphs were kept in separate cages for eclosion. The *C. lividipennis* were checked daily for emergence and supplied with sufficient eggs of *N. lugens*.

### RNA isolation and Illumina sequencing

For transcriptome analysis, approximately 300 pairs of adult antennae from each gender were individually dissected and frozen in liquid nitrogen immediately, then stored at −80 °C till to the RNA isolation. Total RNA was isolated using Trizol reagent (Invitrogen, Carlsbad, CA, USA) following the manufacturer’s protocol. RNA degradation and contamination was monitored on 1% agarose gels. The purity and concentration of RNA were measured using NanoPhotometer^®^ spectrophotometer (IMPLEN, CA, USA) and Qubit^®^ 2.0 Flurometer (Life Technologies, CA, USA). RNA integrity was further assessed using the Agilent Bioanalyzer 2100 system (Agilent Technologies, CA, USA). cDNA library construction and Illumina sequencing for antennae samples were performed at Novogene (Beijing, China). A total amount of 1.5 µg RNA per sample was used, and sequencing libraries were generated using NEBNext^®^ Ultra^TM^ RNA Library Prep Kit for Illumina^®^ (NEB, USA) following manufacturer’s instructions. Briefly, poly-T oligo-attached magnetic beads were used to purify mRNA from total RNA. Fragmentation was carried out using divalent cations under elevated temperature in fragmentation buffer. First strand cDNA was synthesized using random hexamer primer, followed by second strand cDNA synthesis using DNA Polymerase I and RNase H. Remaining overhangs were converted into blunt ends via exonuclease/polymerase activities. After end-repair and ligation of adaptors, the products were amplified by PCR and purified with AMPure XP system (Beckman Coulter, Beverly, USA). The library quality was assessed on the Agilent Bioanalyzer 2100 system. Then the two libraries created from the antennae of male and female *C. lividipennis* were sequenced on an Illumina Hiseq platform and paired-end reads were generated.

### Transcriptome assembly and functional annotation

Raw data (raw reads) of fastq format were firstly processed through in-house perl scripts. In this step, clean data (clean reads) were obtained by removing reads containing adapter, reads containing ploy-N and low quality reads from raw data. At the same time, Q20, Q30, GC-content and sequence duplication level of the clean data were calculated. All the downstream analyses were based on clean data with high quality. The transcriptome *de novo* assembly was performed with Trinity (Grabherr *et al*., 2011) with min_kmer_cov set to 2 by default and all other parameters set default. After assembling, the unigenes were searched against protein databases, such as Nr, Swiss-Prot, KEGG, and GOG, using BLASTx with a cut-off E-value of 10^−5^. Gene Orthology (GO) and Cluster of Orthologous Groups (COG) were determined using Blast2GO program.

### Identification of candidate genes involved in chemoreception

To identify putative OBP, CSP, NPC2, OR, IR, GR and SNMP genes, we searched the transcriptome data set with keywords (odorant-binding protein, chemosensory protein, NPC2, odorant receptor, ionotropic receptor, gustatory receptor and sensory neuron membrane protein). The open reading frames (ORFs) of each unigene was predicted by ORF Finder (https://www.ncbi.nlm.nih.gov/orffinder/). The signal peptides of candidate OBP and CSP genes were predicted using signalP 4.1 (http://www.cbs.dtu.dk/services/SignalP/). In addition, transmembrane domains in proteins (OR and IR) were predicted using TMHMM Server v. 2.0 (http://www.cbs.dtu.dk/services/TMHMM/). To obtain a more reliable sequence, we performed PCR reaction to amplify the intact or partial sequences of each gene. Gene-specific primers were designed online by Primer3 (version 0.4.0) (http://bioinfo.ut.ee/primer3-0.4.0/) based on the transcriptome data (Supplementary Table S4). PCR products were sequenced by a commercial company (Sunny, China).

### Phylogenetic analysis

The phylogenetic analysis was performed based on the amino sequences of the *C. lividipennis* and other insect species olfaction genes. GenBank accession numbers of genes were listed in Supplementary Table S5. The putative amino acid sequences from *C. lividipennis* OBPs, CSPs (without signal peptide sequences) and ORs were aligned using Clustal Omega (https://www.ebi.ac.uk/Tools/msa/clustalo/). We constructed phylogenetic trees using the maximum likelihood analysis with MEGA 7 (JTT model, 1000 bootstrap replications)^65^.

### Relative transcript accumulation of chemosensory genes in female and male antennae

To compare the expression patterns of chemosensory genes in male and female antennae of *C. lividipennis*, qRT-PCR was performed using RNA (3 replicates) extracted from female, male antennae and legs (male and female mixture). Legs were used as the control. PrimerScript RT reagent Kit with gDNA Eraser (Takara, Japan) was used to synthesize cDNA. All the primers used in qRT-PCR were designed online (http://bioinfo.ut.ee/primer3-0.4.0/), and sequences were listed in Supplementary Table S6. The 18S rRNA gene and ribosomal protein S15 (RPS15) were used as reference genes. SYBR Premix Ex Taq II was used in qRT-PCR according to the manufacturer’s protocol. The reaction program was (1) 95 °C, 30 s; (2) 95 °C, 5 s; (3) 60 °C, 30 s; (4) go to (2), 40 cycles in the CFX96 machine (Bio-Rad, Japan). Relative transcript accumulation of different samples were measured by the 2^−ΔΔCt^ method^66^.

### RNA interference (RNAi) targeting *orco*

We performed RNAi to explore the role of *orco* in the foraging behavior of the predator *C. lividipennis*. The MEGAscript T7 High Yield Transcription Kit (Ambion, Austin, TX, USA) was used to synthesize dsRNA. dsRNA primers (Supplementary Table S6) were designed by SnapDragon (http://www.flyrnai.org/cgi-bin/RNAi_find_primers.pl#userconsent#). *Aequorea victoria* green fluorescent protein *(GFP)* was used as the control. We injected 100 fifth instar (day 1) with about 150 ng dsRNA (ds*orco* and ds*GFP*) according a method reported in *N. lugens*^67,68^. Each treatment was replicated three times. Two days after the injections, RNA was extracted from 5 nymphs to examine the gene silencing efficiency by qRT-PCR. The remaining nymphs were used for the olfactory response experiments.

**Figure 6:**
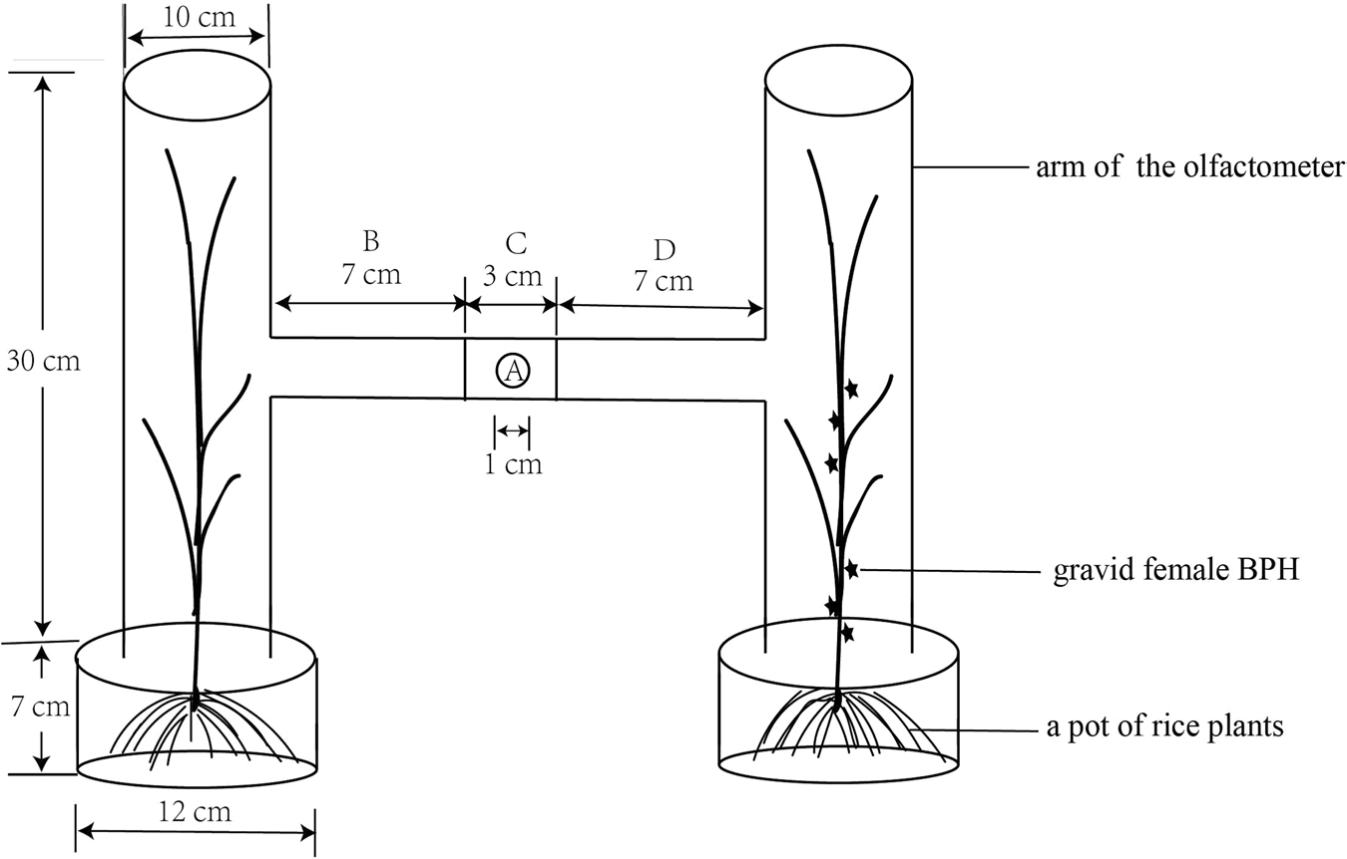
The H-shaped olfactometer used for exploring the responses of *C. lividipennis* to odors after dsRNA treatment. (**A**) Release hole. (**B**) The area that mirids respond to the left odor source. (**C**) The area that mirids do not respond. (**D**) The area that respond to the right odor source.

We tested the responses of *C. lividipennis* treated with ds*orco* and ds*GFP* to different odor sources (healthy plants and plant-BPH gravid female complex) in a two-choice H-shaped olfactometer (Fig. 6), which was similar to the method described by Khan and Saxena^6,69^. The plants used were 40-day-old TN1 rice seedlings. Six rice seedlings were infested by 120 gravid BPH females for 6 h before an assay. 25 fifth instar mirid nymphs (two days after injection) that had been starved for 12 h were introduced into the H-shaped olfactometer through A (1 cm diameter). Two hours later, the number of the predators in the B, C and D area of the glass tube was recorded. The predators that in the C area were regarded as no response mirids. The experiments were conducted in a separate dark room at 26 °C ± 2 °C and 70–80% relative humidity with five biological replicates.

### Statistical analysis

Statistical analysis was performed using Data Processing System (DPS) software v9.5^70^. Data was represented as mean ± SE. Means were compared using two-samples *t* test in choice test of *C. lividipennis*. Relative transcript accumulation of chemosensory genes in female and male antennae was measured by one -way analysis of variance (ANOVA) with the least significant difference (LSD).

## Electronic supplementary material

Supplementary Information

## Data Availability

All data generated or analysed during this study are included in this published article (and its Supplementary Information files).
