# Supplementary material for: Identification and expression analysis of putative chemoreception genes from Cyrtorhinus lividipennis (Hemiptera: Miridae) antennal transcriptome
Source: Sci Rep. 2018 Aug 28;8:12981. doi: 10.1038/s41598-018-31294-9 (PMC6113244; doi:10.1038/s41598-018-31294-9)
Supplement: Supplementary file 1 — Supplementary Information [file 41598_2018_31294_MOESM1_ESM.pdf]

Supplementary Information for

Identification and expression analysis of putative chemoreception genes from *Cyrtorhinus lividipennis* (Hemiptera: Miridae) antennal transcriptome

Gui-Yao Wang, Jing-Lei Zhu, Wen-Wu Zhou, Su Liu, Quais Md Khairul, Naved Ahmad Ansari, Zeng-Rong Zhu*

State Key Laboratory of Rice Biology; Key Laboratory of Molecular Biology of Crop Pathogens and Insects, Ministry of Agriculture; Institute of Insect Sciences, Zhejiang University, Hangzhou, Zhejiang, 310058, China

Email addresses:

Gui-Yao Wang: wangguiyao@yeah.net

Jing-Lei Zhu: 21716101@zju.edu.cn

Wen-Wu Zhou: wenwuzhou@zju.edu.cn

Su Liu: suliu@ahau.edu.cn

Quais Md Khairul: quaisbau@yahoo.com

Naved Ahmad Ansari: navedahmad321@yahoo.com

Zeng-Rong Zhu: zrzhu@zju.edu.cn

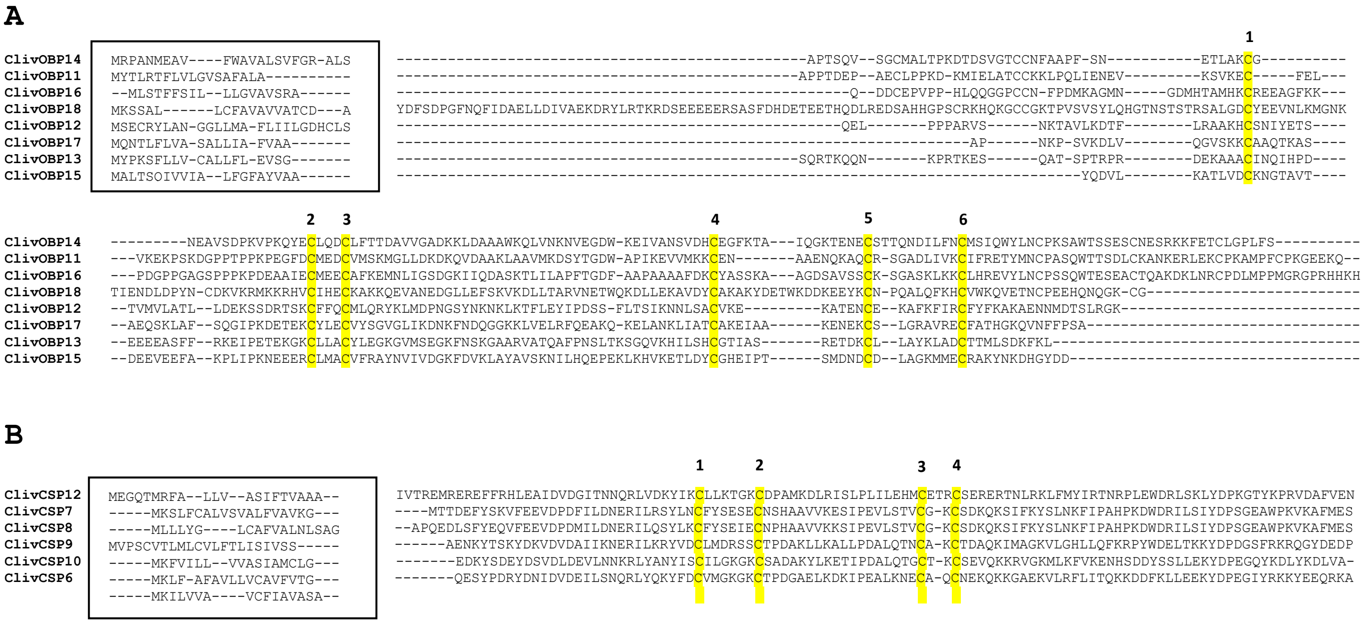

Supplementary Figure S1. Multiple sequence alignment of *C. lividipennis* OBPs (A) and *C. lividipennis* CSPs (B). The predicted signal peptide (inside the box) were depicted separately from the sequences of the native proteins. The conserved cysteine residues were in yellow bands and marked by Arabic numbers 1–6.

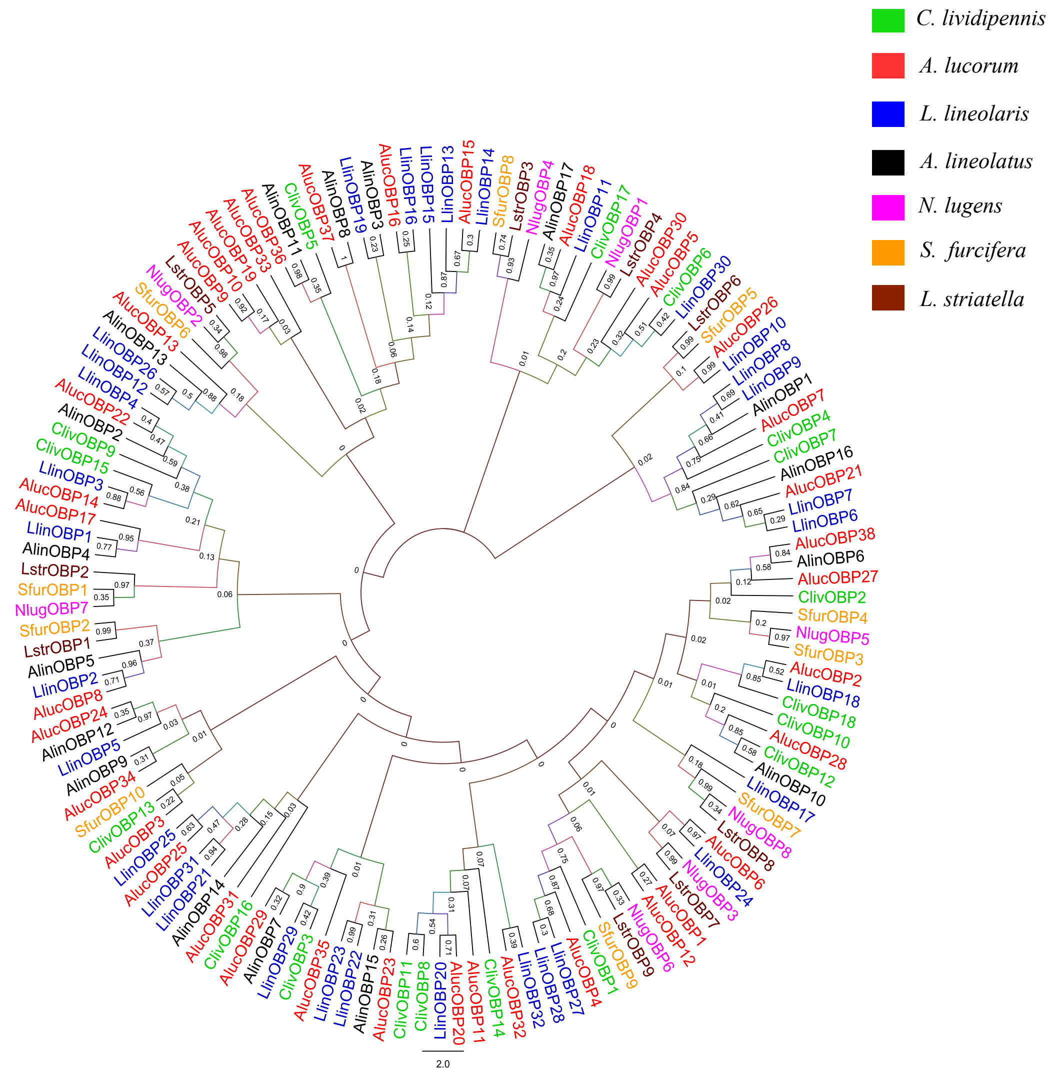

Supplementary Figure S2. Phylogenetic analysis of 132 OBPs from various hemipteran insects. Cliv, *Cyrtorhinus lividipennis*; Aluc, *Apolygus lucorum*; Llin, *Lygus lineolaris*; Alin, *Adelphocoris lineolatus*; Nlug, *Nilaparvata lugens*; Sfur, *Sogatella furcifera*; Lstr, *Laodelphax striatella.*

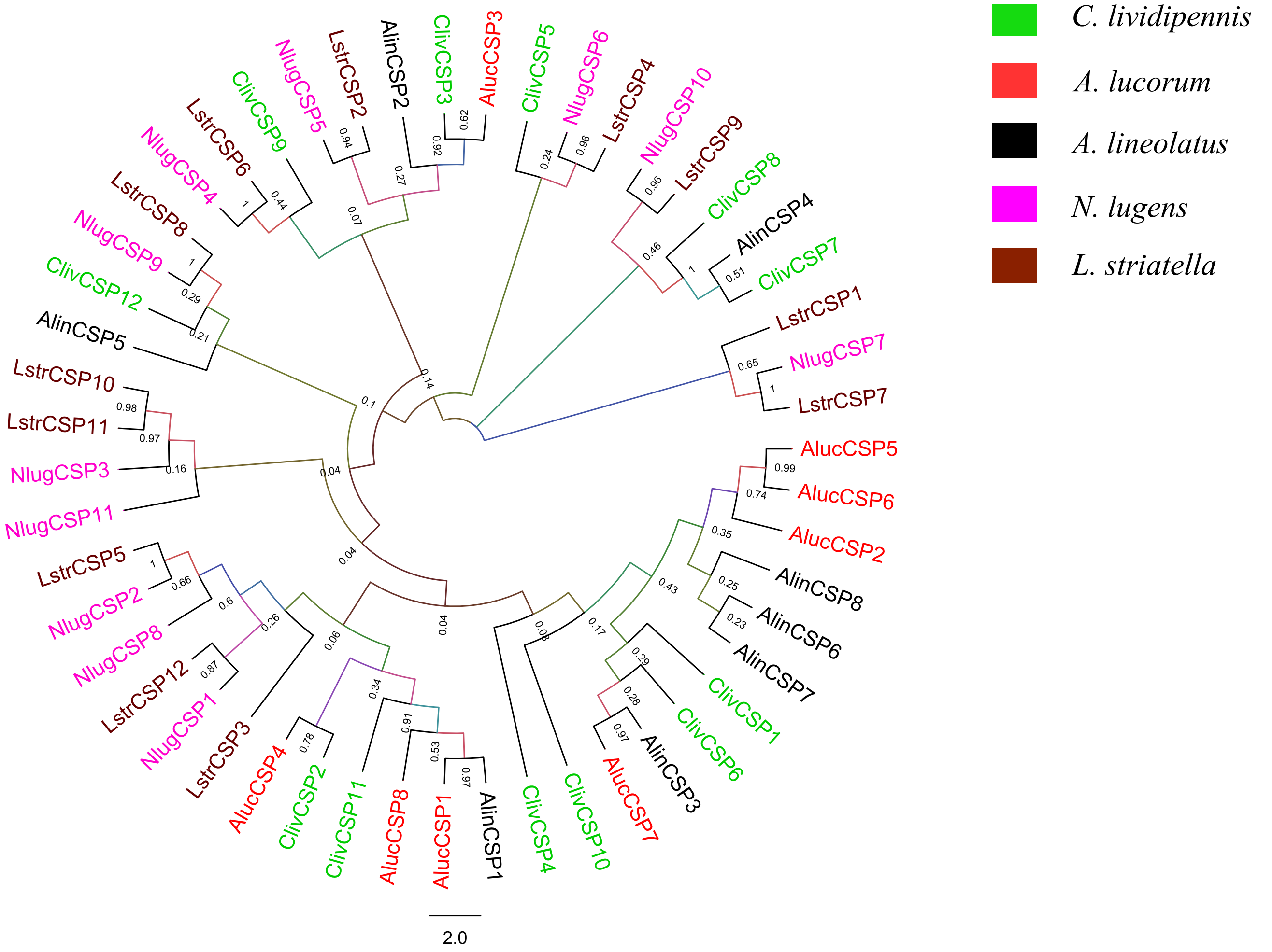

Supplementary Figure S3. Phylogenetic analysis of CSPs from hemipteran insects. Cliv, *Cyrtorhinus lividipennis*; Aluc, *Apolygus lucorum*; Alin, *Adelphocoris lineolatus*; Nlug, *Nilaparvata lugens*; Lstr, *Laodelphax striatella*.

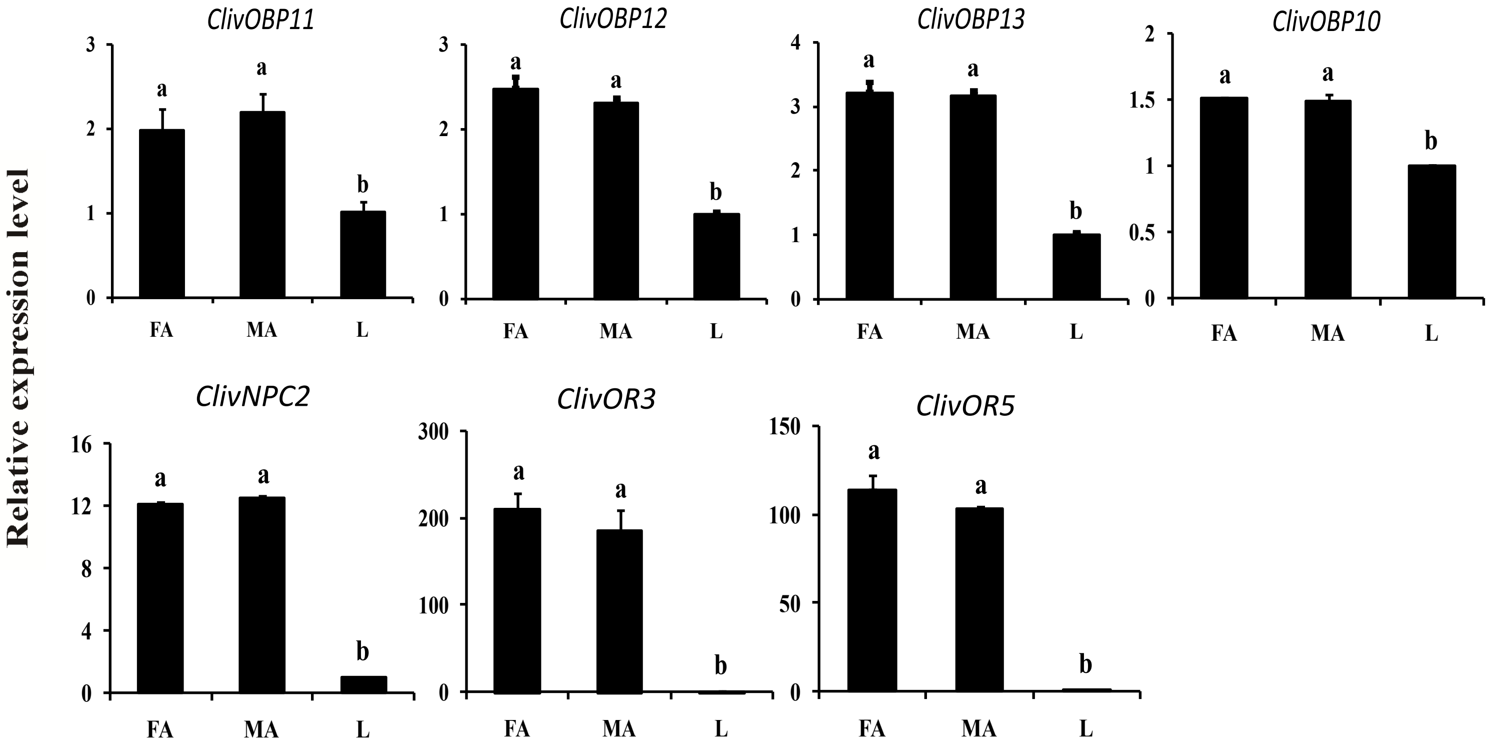

Supplementary Figure S4. The chemosensory genes that showed similar transcript levels in both male and female antennae. Gene expression patterns in antennae were normalized relative to legs (male and female mixture). Data were presented as the mean of three replicates (n=3) ± SE. Different lower cases indicate significant differences ($p<0.05$). FA: female antennae, MA: male antennae, L: legs.

Supplementary Table S1. Overview of the sequencing and assembly process.

	MA (male)	FA (female)
Total Raw Reads	62,553,216	55,283,884
Total Clean Reads	60,658,602	53,853,286
Clean Reads GC (%)	42.02	42.08
Clean Reads Q20 (%)	96.73	96.70
Clean Reads Q30 (%)	91.97	91.91
Combined Trinity assembly of the male and female antennal transcriptomes		
Total Unigenes Number	62,637	
Unigene Min Length (bp)	201	
Unigene Max length (bp)	36,074	
Unigene Mean Length (bp)	1,401	
Unigene N50	2,338	
Unigene N90	588	

Supplementary Table S2. Summary of *OBP*, *CSP* and *NPC2* genes identified in *C. lividipennis.*

Gene name	Acc. no.	ORF (aa)	SP (aa)	Full length	BLASTX best hit				
					Gene name	Species	Acc. no.	E value	Identity (%)
ClivOBP11	MG751808	194	18	Yes	*OBP20*	*Apolygus lucorum*	AMQ76473	4.00E-51	49
ClivOBP12	MG751809	162	26	Yes	*OBP10*	*Adelphocoris lineolatus*	ACZ58081	2.00E-51	59
ClivOBP13	MG751810	154	19	Yes	*OBP34*	*Apolygus lucorum*	AMQ76487	3.00E-64	67
ClivOBP14	MG751811	185	23	Yes	*OBP11*	*Apolygus lucorum*	AFJ54052	2.00E-60	47
ClivOBP15	MG751812	139	20	Yes	*OBP3*	*Lygus lineolaris*	AHF71030	1.00E-72	98
ClivOBP16	MG751813	202	18	Yes	*OBP25*	*Lygus lineolaris*	AHF71056	1.00E-27	44
ClivOBP17	MG751814	145	19	Yes	*OBP11*	*Lygus lineolaris*	AHF71038	5.00E-61	75
ClivOBP18	MG751815	248	19	Yes	*OBP18a*	*Lygus lineolaris*	AHF71046	3.00E-66	72
ClivCSP6	MG751817	131	18	Yes	*CSP6*	*Adelphocoris lineolatus*	ACZ58024	8.00E-54	71
ClivCSP7	MG751818	126	19	Yes	*CSP4*	*Adelphocoris suturalis*	ANA10246	1.00E-65	81
ClivCSP8	MG751819	127	18	Yes	*CSP4*	*Adelphocoris suturalis*	ANA10246	4.00E-62	75
ClivCSP9	MG751820	141	22	Yes	*CSP14*	*Oedaleus asiaticus*	ATI99853	3.00E-30	57
ClivCSP10	MG751821	120	17	Yes	*CSP2*	*Apolygus lucorum*	AGD80082	5.00E-35	50
ClivCSP11	MG751822	127	16	Yes	*CSP3*	*Apolygus lucorum*	AEP95757	9.00E-67	76
ClivCSP12	MG751823	157	21	Yes	*CSP9*	*Adelphocoris lineolatus*	AMD02858	2.00E-33	80
ClivNPC2	MH510335	156	24	Yes	*NPC2 homolog isoform X1*	*Vollenhovia emeryi*	XP_011862436.1	7.00E-35	40

Acc. no.: Accession number; ORF: open reading frame; SP: signal peptides; aa: amino acids.

Supplementary Table S3. Summary of *OR*, *IR*, *GR* and *SNMP* genes identified in *C. lividipennis*.

Gene name	Acc. no.	ORF (aa)	TMD.	Full length	BLASTX best hit				
					Gene name	Species	Acc. no.	E value	Identity (%)
ClivOR1	MG770189	410	6	Yes	*OR60*	*Apolygus lucorum*	AQM56066	1.00E-82	47
ClivOR2	MG770190	314	4	Yes	*OR43*	*Apolygus lucorum*	AQM56049	7.00E-91	43
ClivOR3	MG770191	416	5	Yes	*OR51*	*Apolygus lucorum*	AQM56057	3.00E-162	55
ClivOR4	MG770192	379	5	Yes	*OR61*	*Apolygus lucorum*	AQM56067	4.00E-94	77
ClivOR5	MG770193	375	6	Yes	*OR34*	*Apolygus lucorum*	AQM56040	1.00E-112	56
ClivOR6	MG770194	391	8	Yes	*OR99*	*Apolygus lucorum*	AQM56105	8.00E-119	43
ClivOR7	MG770195	420	5	Yes	*OR7*	*Apolygus lucorum*	AQM56015	8.00E-97	40
ClivOR8	MG770196	435	6	Yes	*OR52*	*Apolygus lucorum*	AQM56058	6.00E-41	28
ClivOR9	MG770197	434	6	Yes	*OR85*	*Apolygus lucorum*	AQM56091	1.00E-110	45
ClivOR10	MG770198	442	5	Yes	*OR62*	*Apolygus lucorum*	AQM56068	2.00E-91	63
ClivOR11	MG770199	399	6	Yes	*OR40*	*Apolygus lucorum*	ANC27967	1.00E-166	57
ClivOR12	MG770200	391	3	Yes	*OR67*	*Apolygus lucorum*	AQM56073	1.00E-144	53
ClivOR13	MG770201	423	6	Yes	*OR17*	*Apolygus lucorum*	AQM56025	4.00E-134	45
ClivOR14	MG770202	401	4	Yes	*OR100*	*Apolygus lucorum*	AQM56106	3.00E-162	55
ClivORCO	MG770203	490	6	Yes	*ORCO*	*Adelphocoris lineolatus*	AHC72292	0	89
ClivIR1	MG770204	244	2	No	*IR40a*	*Nilaparvata lugens*	XP_022197172	6.00E-93	57
ClivIR2	MG770205	419	3	Yes	*IR41a*	*Ostrinia furnacalis*	BAR64800	3.00E-58	31
ClivIR3	MG770206	662	2	Yes	*IR*	*Eogystia hippophaecolu*	AOG12846	4.00E-63	30
ClivIR4	MG770207	552	2	Yes	*IR64a*	*Ostrinia furnacalis*	BAR64801	3.00E-24	22
ClivIR5	MG770208	198	1	No	*IR24*	*Locusta migratoria*	ALD51351	8.00E-25	38
ClivIR6	MG770209	504	3	Yes	*IR40a isoform X2*	*Myzus persicae*	XP_022182311	7.00E-163	48
ClivGR1	MH510328	364	8	Yes	*GR121*	*Tribolium castaneum*	EFA07618.1	5.00E-05	26
ClivGR2	MH510329	355	7	Yes	*GR3*	*Nasonia vitripennis*	NP_001164386.1	2.00E-05	28
ClivGR3	MH510330	362	7	Yes	*GR for sugar taste 43a-like*	*Nilaparvata lugens*	XP_022196424.1	9.00E-09	28
ClivSNMP1	MH510331	557	2	Yes	*SNMP 1-like*	*Nilaparvata lugens*	XP_022198804.1	4.00E-158	51
ClivSNMP2-1	MH510333	560	2	Yes	*SNMP 2-like*	*Athalia rosae*	XP_012267137.1	4.00E-91	36
ClivSNMP2-2	MH510334	507	2	Yes	*SNMP 2-like isoform X1*	*Nilaparvata lugens*	XP_022190215.1	1.00E-94	35

Acc. no.: Accession number; ORF: open reading frame; TMD: transmembrane domains; aa: amino acids.

Supplementary Table S4: Primers used in PCR assays.

Gene	Direction	Sequence (5'-3')	Product (bp)
CLOBP11	F	TTTTTGGTCCTTGGCGTATC	529
	R	CTTTTGGGCACTTTTCCAAC	
CLOBP12	F	GGCCTTCTAATGGCTTTCCT	456
	R	TTTTCCTCGAAGCGAAGTGT	
CLOBP13	F	CTCGTGTGCGCTCTTCTTTT	410
	R	CAATCCGCTAGCTTGTAGGC	
CLOBP14	F	CGGCTAACATGGAAGCTGTT	512
	R	CCTCGACTCATTGCAGGACT	
CLOBP15	F	GCTTTGACGTCCCAAATTGT	410
	R	TCATAACCGTGGTCTTTGTTG	
CLOBP16	F	CTTTTATTGGGCGTTGCTGT	505
	R	TGCATGCCTCACTTTCAGTC	
CLOBP17	F	GGCACTCCTCATTGCCTTC	402
	R	CTGGGGAAGAAGTTGACCTG	
CLOBP18	F	ACCTGCGATGCTTACGACTT	635
	R	TGCTTGAATTGGAGTGCTTG	
CLCSP6	F	CACGGGCCAAGAATCATATC	319
	R	CGCTTTTCTCTGCTCTTCGT	
CLCSP7	F	TGGCGCTATTTGTTGCTGTA	308
	R	TGATGGGTCGTAAATGGACA	
CLCSP8	F	CCGCAAGAGGATTTGTCATT	320
	R	TCCATGAAGGCTTTCACTTTG	
CLCSP9	F	TCCGTCCTGTGTCACTCTCA	418
	R	CGGTCTTGAAGAGTCGTCGT	
CLCSP10	F	TTCGATCGCTATGTGTCTCG	303
	R	TATTGTCCTTCGGGGTCGTA	
CLCSP11	F	CATCGACCTCGAAGAGATCC	301
	R	GGGAAGTTTGATTCCACGTT	
CLCSP12	F	CTTTTGGTTGCCAGCATCTT	419
	R	GACCGGTTGATTGTCGGTAG	

CLOR1	F	ATGGAAGACGACCCAGACAG	801
	R	TTGGCTGATCCAATACGTGA	
CLOR2	F	CCGTACTCAGAACACGCTGA	800
	R	TCCTCTTCACCGTTGACAATC	
CLOR3	F	AACACTTCCTTGCTCGCCTA	829
	R	TGGCGAGGAAAATGAAGAAG	
CLOR4	F	CCCGAGTAAATTCTCGACCA	845
	R	CGAATGTCGAGTTTGGGTCT	
CLOR5	F	CGTGTCCTCCAATCAACCTT	897
	R	ACGTGAAGCCAGTTGCTTTT	
CLOR6	F	CATGTCAACGGTCAATCGAG	813
	R	AACATTTTCACGCCTTGTCC	
CLOR7	F	AATTGGCCGCTCTTTATCCT	847
	R	ATAAGAGCTCCGCCATCAAA	
CLOR8	F	TGAGGACAACGTGGATTTGA	843
	R	TCCCCGAAGTAACTGACCAC	
CLOR9	F	ACTTAGGACAGGCTGCCAGA	821
	R	CATGCAGAGCAGAAGAGCTG	
CLOR10	F	CTGATCGGAGTGATCGGATT	843
	R	AGCGGTCAGCATGCTAAAAT	
CLOR11	F	TCGGCTCGTCTTTCAAGTTT	811
	R	AGCAAAAGCTAAGCCAACCA	
CLOR12	F	CAGTGGTCACCAACGATTTG	818
	R	TTTCAACGTCCCTTCATTCC	
CLOR13	F	CCTAGTCGCAATGGGTTGTT	862
	R	GGGCCATAAGTACCATCGTG	
CLOR14	F	CACGCTCTGGATCGTCTACA	834
	R	AATCGAGAAACGAGCCTTGA	
CLORCO	F	GAAATACCGCAGCTCATGGT	817
	R	AAAATTTGGCTCCTGACACG	
CLIR1	F	ATGAGAGAAGCTTGGCCGTA	633
	R	ATGTTTCCCTTCCTCCCAAT	
CLIR2	F	TCTTCCCTTACATGCCATCC	851
	R	GTGTTCGTTGGACCTGGATT	
CLIR3	F	TGCCGTACGGTAACAATGAA	818

	R	TATTCCAAGGCCGATGCTAC	
CLIR4	F	CCTCTCTTGCCTGAAAAACG	864
	R	ATCAGCGACAAGCCAGCTAT	
CLIR5	F	CAAATTTTGGCGACGCTATT	505
	R	AGATCATGCTCCGAAAATGG	
CLIR6	F	ATGAGAGAAGCTTGGCCGTA	856
	R	ATGGGAGTCTCTCCGGTTTT	
CLGR1	F	ACATCCTTCACCGTTGCTTC	806
	R	GAAACCACAAGCGGTGAAAT	
CLGR2	F	CCTTTGTTCGAGGAAAGCAA	811
	R	CCACAATGAACATCGTCGTC	
CLGR3	F	CACCTCCTATCTTGCGCTTC	850
	R	ATGTGACCAAGGGGTAACCA	
CLNPC2	F	TTTGTGCTGGGATCCTGATA	408
	R	CCTCGCTCTGTTCATCCTCT	
CLSNMP1-1	F	GGCCAAATTCCCAATATCCT	1141
	R	GTGCAATCCTTCGACCTCAT	
CLSNMP2-1	F	TTTCCTAAACAACGCCATCC	923
	R	GCCCGATGATGTGGTAGAGT	
CLSNMP2-2	F	ACCAGAAGTCATGGGCAAAG	982
	R	GCCAACGAGTAGCATCAACA	

Supplementary Table S5: GenBank accession numbers of insect *OBPs*, *CSPs* and *ORs* used in the phylogenetic tree.

Gene	Acc. no.	Gene	Acc. no.	Gene	Acc. no.
Cyrtorhinus lividipennis					
ClivOBP1	KY462016	*ClivOBP7*	KY462022	*ClivOBP13*	MG751810
ClivOBP2	KY462017	*ClivOBP8*	KY462023	*ClivOBP14*	MG751811
ClivOBP3	KY462018	*ClivOBP9*	KY462024	*ClivOBP15*	MG751812
ClivOBP4	KY462019	*ClivOBP10*	KY462025	*ClivOBP16*	MG751813
ClivOBP5	KY462020	*ClivOBP11*	MG751808	*ClivOBP17*	MG751814
ClivOBP6	KY462021	*ClivOBP12*	MG751809	*ClivOBP18*	MG751815
ClivCSP1	KY462026	*ClivCSP5*	KY462030	*ClivCSP9*	MG751820
ClivCSP2	KY462027	*ClivCSP6*	MG751817	*ClivCSP10*	MG751821
ClivCSP3	KY462028	*ClivCSP7*	MG751818	*ClivCSP11*	MG751822
ClivCSP4	KY462029	*ClivCSP8*	MG751819	*ClivCSP12*	MG751823
ClivOR1	MG770189	*ClivOR6*	MG770194	*ClivOR11*	MG770199
ClivOR2	MG770190	*ClivOR7*	MG770195	*ClivOR12*	MG770200
ClivOR3	MG770191	*ClivOR8*	MG770196	*ClivOR13*	MG770201
ClivOR4	MG770192	*ClivOR9*	MG770197	*ClivOR14*	MG770202
ClivOR5	MG770193	*ClivOR10*	MG770198	*ClivORCO*	MG770203
Apolygus lucorum					
AlucOBP1	AEA07705	*AlucOBP14*	AMQ76467	*AlucOBP27*	AMQ76480
AlucOBP2	AEA07706	*AlucOBP15*	AMQ76468	*AlucOBP28*	AMQ76481
AlucOBP3	AEA07661	*AlucOBP16*	AMQ76469	*AlucOBP29*	AMQ76482
AlucOBP4	AEA07662	*AlucOBP17*	AMQ76470	*AlucOBP30*	AMQ76483
AlucOBP5	AEA07663	*AlucOBP18*	AMQ76471	*AlucOBP31*	AMQ76484
AlucOBP6	AEA07664	*AlucOBP19*	AMQ76472	*AlucOBP32*	AMQ76485
AlucOBP7	AFJ54048	*AlucOBP20*	AMQ76473	*AlucOBP33*	AMQ76486
AlucOBP8	AFJ54049	*AlucOBP21*	AMQ76474	*AlucOBP34*	AMQ76487
AlucOBP9	AFJ54050	*AlucOBP22*	AMQ76475	*AlucOBP35*	AMQ76488
AlucOBP10	AFJ54051	*AlucOBP23*	AMQ76476	*AlucOBP36*	AMQ76489

AlucOBP11	AFJ54052	*AlucOBP24*	AMQ76477	*AlucOBP37*	AMQ76490
AlucOBP12	AFJ54053	*AlucOBP25*	AMQ76478	*AlucOBP38*	AMQ76491
AlucOBP13	AMQ76466	*AlucOBP26*	AMQ76479		
AlucCSP1	AGD80081	*AlucCSP4*	AGD80084	*AlucCSP7*	AGD80087
AlucCSP2	AGD80082	*AlucCSP5*	AGD80085	*AlucCSP8*	AGD80088
AlucCSP3	AGD80083	*AlucCSP6*	AGD80086		
AlucORCO	AHC72290	*AlucOR88*	AQM56094	*AlucOR65*	AQM56071
AlucOR46	ANE06404	*AlucOR87*	AQM56093	*AlucOR63*	AQM56069
AlucOR107	AQM56113	*AlucOR86*	AQM56092	*AlucOR59*	AQM56065
AlucOR106	AQM56112	*AlucOR85*	AQM56091	*AlucOR58*	AQM56064
AlucOR105	AQM56111	*AlucOR84*	AQM56090	*AlucOR57*	AQM56063
AlucOR103	AQM56109	*AlucOR81*	AQM56087	*AlucOR56*	AQM56062
AlucOR100	AQM56106	*AlucOR79*	AQM56085	*AlucOR54*	AQM56060
AlucOR99	AQM56105	*AlucOR78*	AQM56084	*AlucOR52*	AQM56058
AlucOR97	AQM56103	*AlucOR75*	AQM56081	*AlucOR51*	AQM56057
AlucOR96	AQM56102	*AlucOR73*	AQM56079	*AlucOR50*	AQM56056
AlucOR94	AQM56100	*AlucOR71*	AQM56077	*AlucOR48*	AQM56054
AlucOR93	AQM56099	*AlucOR70*	AQM56076	*AlucOR47*	AQM56053
AlucOR90	AQM56096	*AlucOR68*	AQM56074	*AlucOR45*	AQM56051
AlucOR89	AQM56095	*AlucOR67*	AQM56073	*AlucOR43*	AQM56049
AlucOR22	AQM56030	*AlucOR23*	AQM56031	*AlucOR42*	AQM56048
AlucOR21	AQM56029	*AlucOR25*	AQM56033	*AlucOR40*	AQM56046
AlucOR20	AQM56028	*AlucOR27*	AQM56035	*AlucOR39*	AQM56045
AlucOR17	AQM56025	*AlucOR29*	AQM56036	*AlucOR37*	AQM56043
AlucOR16	AQM56024	*AlucOR31*	AQM56037	*AlucOR36*	AQM56042
AlucOR14	AQM56022	*AlucOR32*	AQM56038	*AlucOR34*	AQM56040
AlucOR13	AQM56021	*AlucOR5*	AQM56013	*AlucOR30*	AKS44363
AlucOR11	AQM56019	*AlucOR4*	AQM56012	*AlucOR28*	AKS44362
AlucOR10	AQM56018	*AlucOR3*	AQM56011	*AlucOR18*	AKS44361
AlucOR9	AQM56017	*AlucOR2*	AQM56010	*AlucOR12*	AKS44360

AlucOR7	AQM56015	*AlucOR1*	AQM56009		
Adelphocoris lineolatus					
AlinOBP1	ACZ58027	*AlinOBP7*	ACZ58085	*AlinOBP13*	ACZ580840
AlinOBP2	ACZ58028	*AlinOBP8*	ACZ58079	*AlinOBP14*	ACZ58086
AlinOBP3	ACZ58029	*AlinOBP9*	ACZ58080	*AlinOBP15*	AMD02855
AlinOBP4	ACZ58030	*AlinOBP10*	ACZ58081	*AlinOBP16*	AMD02856
AlinOBP5	ACZ58031	*AlinOBP11*	ACZ58082	*AlinOBP17*	AMD02857
AlinOBP6	ACZ58032	*AlinOBP12*	ACZ58083		
AlinCSP1	ACZ58019	*AlinCSP4*	ACZ58022	*AlinCSP7*	ACZ58025
AlinCSP2	ACZ58021	*AlinCSP5*	ACZ58023	*AlinCSP8*	ACZ58026
AlinCSP3	ACZ58020	*AlinCSP6*	ACZ58024		
Lygus lineolaris					
LlinOBP1	AHF71028	*LlinOBP12*	AHF71039	*LlinOBP23*	AHF71053
LlinOBP2	AHF71029	*LlinOBP13*	AHF71040	*LlinOBP24*	AHF71055
LlinOBP3	AHF71030	*LlinOBP14*	AHF71041	*LlinOBP25*	AHF71056
LlinOBP4	AHF71031	*LlinOBP15*	AHF71042	*LlinOBP26*	AHF71057
LlinOBP5	AHF71032	*LlinOBP16*	AHF71044	*LlinOBP27*	AHF71058
LlinOBP6	AHF71033	*LlinOBP17*	AHF71045	*LlinOBP28*	AHF71059
LlinOBP7	AHF71034	*LlinOBP18*	AHF71046	*LlinOBP29*	AHF71060
LlinOBP8	AHF71035	*LlinOBP19*	AHF71049	*LlinOBP30*	AHF71061
LlinOBP9	AHF71036	*LlinOBP20*	AHF71050	*LlinOBP31*	AHF71062
LlinOBP10	AHF71037	*LlinOBP21*	AHF71051	*LlinOBP32*	AHF71063
LlinOBP11	AHF71038	*LlinOBP22*	AHF71052		
Nilaparvata lugens					
NlugOBP1	ACI30679	*NlugOBP4*	AGZ04895	*NlugOBP7*	AGZ04898
NlugOBP2	ACI30680	*NlugOBP5*	AGZ04896	*NlugOBP8*	AGZ04899
NlugOBP3	ACI30681	*NlugOBP6*	AGZ04897		
NlugCSP1	ASL05006	*NlugCSP5*	ASL04982	*NlugCSP9*	ASL05050
NlugCSP2	ASL05045	*NlugCSP6*	ASL05030	*NlugCSP10*	ASL05051
NlugCSP3	ASL05046	*NlugCSP7*	ASL05048	*NlugCSP11*	ASL05052

NlugCSP4	ASL05047	*NlugCSP8*	ASL05049		
NlugORCO	XP_022185660	*NlugOR63a-likee*	XP_022189749	*NlugOR43a-like*	XP_02220474
NlugOR46a	XP_022185545	*NlugOR23a-like*	XP_022199828	*NlugOR56a-like*	XP_02220034
NlugOR83a-like	XP_022200228	*NlugOR43a-likeisoform X2*	XP_022199283		
NlugOROr2-like	XP_022192649	*NlugOR7a-like*	XP_022207573		
Sogatella furcifera					
SfurOBP1	AGZ04901	*SfurOBP5*	AGZ04905	*SfurOBP9*	AGZ04909
SfurOBP2	AGZ04902	*SfurOBP6*	AGZ04906	*SfurOBP10*	AGZ04910
SfurOBP3	AGZ04903	*SfurOBP7*	AGZ04907		
SfurOBP4	AGZ04904	*SfurOBP8*	AGZ04908		
Laodelphax striatella					
LstrOBP1	AGZ04920	*LstrOBP4*	AGZ04923	*LstrOBP7*	AGZ04926
LstrOBP2	AGZ04921	*LstrOBP5*	AGZ04924	*LstrOBP8*	AGZ04927
LstrOBP3	AGZ04922	*LstrOBP6*	AGZ04925	*LstrOBP9*	AGZ04928
LstrCSP1	AGZ04929	*LstrCSP5*	AGZ04933	*LstrCSP9*	AGZ04937
LstrCSP2	AGZ04930	*LstrCSP6*	AGZ04934	*LstrCSP10*	AGZ04938
LstrCSP3	AGZ04931	*LstrCSP7*	AGZ04935	*LstrCSP11*	AGZ04939
LstrCSP4	AGZ04932	*LstrCSP8*	AGZ04936	*LstrCSP12*	AGZ04940
Myzus persicae					
MperOR46a-like	XP_022178692	MperOR22c-like	XP_022167641	MperORCOisoformX1	XP_02216289
MperOR2a-like	XP_022175226	MperOR67a-like	XP_022167526	MperOR4-like	XP_02216456
MperOR43b-like	XP_022173211	MperORCOisoformX2	XP_022162893	MperOR33b-like	XP_02216909
Acyrthosiphon pisum					
ApisOR43	AQS60755	ApisOR17	AQS60746	ApisOR31	AQS60750
ApisOR42	AQS60754	ApisOR10	AQS60745	ApisOR25	AQS60749
ApisOR39	AQS60753	ApisOR5	AQS60744	ApisOR23	AQS60748
ApisOR38	AQS60752	ApisOR4	AQS60743	ApisOR20	AQS60747
ApisOR37	AQS60751	ApisOR2	AQS60742	ApisORCO	AQS60741

Supplementary Table S6: Primers used in qPCR and RNAi assays.

Gene	Direction	Sequence (5'-3')	Product (bp)
CLOBP11	F	ACCCCAACTCATTGAAAACG	148
	R	GAGCCCCATTTTGGACATTA	
CLOBP12	F	AGCTCAGATCGAACCTCCAA	136
	R	ACGTCAAAAACGACGAATCC	
CLOBP13	F	AGTAAAGGAGCGGCCAGAGT	146
	R	CAATCCGCTAGCTTGTAGGC	
CLOBP14	F	ACCACCGACGCTGTAGTAGG	149
	R	TTTCCCTGGATTGCTGTCTT	
CLOBP15	F	GCTTTGACGTCCCAAATTGT	139
	R	TGGCGAATTCTTCAACTTCC	
CLOBP16	F	ACAAATGCTACGCCAGTTCC	144
	R	TGCATGCCTCACTTTCAGTC	
CLOBP17	F	GTGTACAGCGGAGTCGGATT	149
	R	TCCTTGGCAGCAATCTCTTT	
CLOBP18	F	ACTGCTCGGGTGAATGAAAC	140
	R	TGCTTGAATTGGAGTGCTTG	
CLCSP6	F	GGACGAAATTTTGAGCAACC	147
	R	TGTTTTTCGTTGCATTGAGC	
CLCSP7	F	CTATCCCCGAAGTGCTCTCA	137
	R	TGATGGGTCGTAAATGGACA	
CLCSP8	F	CCGCAAGAGGATTTGTCATT	144
	R	TTTAACTACGGCAGCGTGTG	
CLCSP9	F	GACAAACTGCGCCAAGTGTA	149
	R	CATCGTAGCCTTGCCTTTTC	
CLCSP10	F	CGGATGCCAAATACCTGAAG	149
	R	CTTCTCCAAGAGGCTGCTGT	
CLCSP11	F	ACAGCAAGAAGGAACGGACA	135
	R	CCACGTTTTTGAGCTTCCTC	
CLCSP12	F	TATTGAAAACCGGGAAGTGC	149
	R	CGGTCGATTCGTCCTTATGT	

CLOR1	F	TTGCTCAAGACGCAGACATC	139
	R	TTTGGACGACTCTGACAACG	
CLOR2	F	ACACGCTGAAGCGAAAATCT	148
	R	CCTCTCACCGAATGCAAAAT	
CLOR3	F	AATGAAATTTGGCGCTCTGT	149
	R	TGGTGAAATGAGCAGACTCG	
CLOR4	F	CCTCGTCCTCCATTCGATTA	142
	R	CGCAATGACGAAGAATCTCA	
CLOR5	F	GCAACATTTTGTGCGATTTG	137
	R	GCATGTCGGTCCTTGATCTT	
CLOR6	F	TTCTTCGCGATAATCGTTCC	138
	R	GCCAACTCCTTTGTGGTCAT	
CLOR7	F	GTGGACGGTCTGAACTGGAT	143
	R	ATAGAGCCCGAACAAGCAAA	
CLOR8	F	CGTTTGTCGGTGGCTAACTT	148
	R	TCTTGATGGCATGGTGATGT	
CLOR9	F	CAATGCACCCATGTTCACTC	142
	R	TCTGGCAGCCTGTCCTAAGT	
CLOR10	F	CGTAGGGTACACGGACGACT	146
	R	TTCCGAGAGCCAACATAAGG	
CLOR11	F	ACAAGCAAATGCTGGGATTC	147
	R	CAGCAAAAGCTAAGCCAACC	
CLOR12	F	GTGTATTTCTGCGTGGAGCA	141
	R	CCGCACAGGTAGAAGAAAGC	
CLOR13	F	AATCGTCATGCAGTTCAACG	149
	R	CGTATGCCCGCTTGTTAAGT	
CLOR14	F	CATGCGAAGCTTACCCTCTC	146
	R	CGCTACGGCCTTATGTTTGT	
CLORCO	F	GCGCATGGAATCAGGTAAAT	139
	R	GTCGTCCAGGAGAAGACAGC	
CLIR1	F	CTCACACATGCTCCTGGAAA	143
	R	TGCCATCCTAACCGAACTTT	
CLIR2	F	TGAGAGCGTCTCTGGAATCA	150
	R	GCGCTGCTATAAATGGTGGT	

CLIR3	F	GTTCGATCGGAATGTTCGTT	150
	R	TCCTCGGGATTGTGAGGTAG	
CLIR4	F	ATAGCTGGCTTGTCGCTGAT	140
	R	TTGATTGGGATGTCGGTTTT	
CLIR5	F	CTGTGCTGGCAACAAGAAAA	147
	R	GCCGTAGATGAGCGCTAAAA	
CLIR6	F	AGCCCTGCAGAGTCATTCAT	146
	R	ATTAAAACGAGCGCTCCAAA	
CLGR1	F	TATTGTATTGGCGGGATCGT	143
	R	GTGGCATTTTCGATTTCGTT	
CLGR2	F	TTCGTTCGCTGATTGAAATG	150
	R	CCACAATGAACATCGTCGTC	
CLGR3	F	AACCAAAAATGCAGGGAATG	144
	R	GCGCAAGATAGGAGGTGAAG	
CLNPC2	F	CTGTGTGGACAGTGGGATCA	137
	R	CCTCGCTCTGTTCATCCTCT	
CLSNMP1	F	TTGGACCGAAAAATGACACA	140
	R	TTTATTGCACTCAGGCGTTG	
CLSNMP2-1	F	GAGCGCAAGTCATTTTCTCC	139
	R	TCGAGAGGTGCTCCTGTTTT	
CLSNMP2-2	F	GTTCCAATCGTGCTGTCCTT	138
	R	TTTTACGGGAACTCCAGTCG	
CL18S	F	CGCTAGAAGTCCGTCAGAGC	132
	R	AAGTGGGAGTCAGGGTCCTT	
CLRPS15	F	GGGTACATTGGGGAGTTTGA	151
	R	GCAGAAGGTTGTTCGTCCAT	
ds*CLOrco*	F	CCTGTATGGGAAAAGACGGA	449
	R	ACTGTTCCTCCGTTCACACC	
ds*GFP*	F	AAGTTCAGCGTGTCCGGCGA	414
	R	CACCTTGATGCCGTTCTTCT	
